# Gene regulatory network inference: evaluation and application to ovarian cancer allows the prioritization of drug targets

**DOI:** 10.1186/gm340

**Published:** 2012-05-01

**Authors:** Piyush B Madhamshettiwar, Stefan R Maetschke, Melissa J Davis, Antonio Reverter, Mark A Ragan

**Affiliations:** 1 The University of Queensland, Institute for Molecular Bioscience, 306 Carmody Road, St Lucia, Brisbane, Queensland 4072, Australia; 2 Australian Research Council Centre of Excellence in Bioinformatics; 3 Queensland Facility for Advanced Bioinformatics, 306 Carmody Road, Brisbane, Queensland 4072, Australia; 4 CSIRO Livestock Industries, 306 Carmody Road, St Lucia, Brisbane, Queensland 4072, Australia

## Abstract

**Background:**

Altered networks of gene regulation underlie many complex conditions, including cancer. Inferring gene regulatory networks from high-throughput microarray expression data is a fundamental but challenging task in computational systems biology and its translation to genomic medicine. Although diverse computational and statistical approaches have been brought to bear on the gene regulatory network inference problem, their relative strengths and disadvantages remain poorly understood, largely because comparative analyses usually consider only small subsets of methods, use only synthetic data, and/or fail to adopt a common measure of inference quality.

**Methods:**

We report a comprehensive comparative evaluation of nine state-of-the art gene regulatory network inference methods encompassing the main algorithmic approaches (mutual information, correlation, partial correlation, random forests, support vector machines) using 38 simulated datasets and empirical serous papillary ovarian adenocarcinoma expression-microarray data. We then apply the best-performing method to infer normal and cancer networks. We assess the druggability of the proteins encoded by our predicted target genes using the CancerResource and PharmGKB webtools and databases.

**Results:**

We observe large differences in the accuracy with which these methods predict the underlying gene regulatory network depending on features of the data, network size, topology, experiment type, and parameter settings. Applying the best-performing method (the supervised method SIRENE) to the serous papillary ovarian adenocarcinoma dataset, we infer and rank regulatory interactions, some previously reported and others novel. For selected novel interactions we propose testable mechanistic models linking gene regulation to cancer. Using network analysis and visualization, we uncover cross-regulation of angiogenesis-specific genes through three key transcription factors in normal and cancer conditions. Druggabilty analysis of proteins encoded by the 10 highest-confidence target genes, and by 15 genes with differential regulation in normal and cancer conditions, reveals 75% to be potential drug targets.

**Conclusions:**

Our study represents a concrete application of gene regulatory network inference to ovarian cancer, demonstrating the complete cycle of computational systems biology research, from genome-scale data analysis via network inference, evaluation of methods, to the generation of novel testable hypotheses, their prioritization for experimental validation, and discovery of potential drug targets.

## Background

Cancer is a disease not of single genes, but rather of genomes [1] and/or networks of molecular interaction and control [2]. Reconstructing gene regulatory networks (GRNs) in healthy and diseased tissues is therefore critical to understanding cancer phenotypes and devising effective therapeutics [3]. Conventional experimental approaches are focused on individual genes and consequently too time-consuming for reverse-engineering the large number of interactions in GRNs. By contrast, system-wide computational approaches can deal with complex networks of interacting molecules [4]. GRNs are typically represented as graphs in which nodes represent genes (for example, encoding a transcription factor or its target gene), and edges their regulatory interaction [3,5-7].

Advances in microarray and, more recently, next-generation sequencing technologies provide a wealth of data for GRN inference (GRNI). Many diverse GRNI methods have been proposed, reflecting the enormous interest in the field, and the richness of computational mathematics, multivariate statistics and information science. These methods can be classified into two categories, unsupervised and supervised [8,9]. In the former, networks are inferred exclusively from the data (for example, differential gene expression), whereas supervised methods require additional knowledge of regulatory interactions as a training set. Unsupervised methods can largely be divided into two groups: those based on correlation and those based on mutual information [10,11]. The former tend to be algorithmically simple and computationally fast but frequently assume linear relationships among variables. In contrast, methods based on mutual information capture non-linear as well as linear interactions but are applicable only to discrete data and need to employ discretization methods, which can be computationally demanding.

Given this diversity, it is critical that users understand the relative strengths and limitations of GRNI methods. To this end, DREAM (Dialogue for Reverse Engineering Assessments and Methods), an annual open competition in network inference, has been established [12]. Gene expression (and other) data, but not the underlying GRNs, are published, and teams apply GRNI technologies to reverse-engineer, as accurately as possible, the underlying network. While overall performance is modest and no clear winning approach is yet apparent, certain important themes have emerged [13-15].

First, GRNI methods perform differently on different types of data. For instance, methods based on linear models perform poorly on highly non-linear data such as may arise from drastic perturbations like gene knockouts, whereas non-linear methods may perform well in these scenarios [16]. Single-point or steady-state data typically yield better predictions than do time-course data [14]. Data size is often critical, with the low accuracies observed on genome-scale networks improved for smaller subsets [13,17]. Less predictably, some methods excel on networks of Erdös-Rényi topology, others on scale-free networks [13].

Second, with the current GRNI methods, simpler approaches (for example, correlation) often outperform more-complicated ones even on synthetic data, presumably because the methodological complications fail to capture key complexities of the underlying models and/or combinatorial regulation [18]. Further, prediction accuracy is usually even lower with real-life data than with simulated data, probably not only because the former tend to be less complete and/or of lower quality, and the underlying networks larger and of unknown topology, but also because actual cellular systems involve layers of regulatory control, including chromatin remodeling, small RNAs and metabolite-based feedback [3], that existing GRNI methods cannot adequately model. Furthermore, tumors are heterogeneous and involve non-standard or unique disruptions or regulatory interactions, rendering GRN inference even more challenging [19].

Various measures of prediction accuracy have been applied, including the F1 score, Matthews correlation coefficient, and area under the receiver-operating characteristic (ROC) curve (AUC) [20]. Each of these measures is expressed as a single numerical value that integrates over all predicted interactions. Yet even a GRN predicted with overall low accuracy may contain a subset of predictions likely to be correct and therefore worthy of subsequent investigation, potentially including experimental validation.

Here we select from about 80 published GRNI methods [21-28] one supervised and eight unsupervised methods that together represent a diversity of mathematical formalisms. Our selection was guided by whether the software is documented, supported and could be installed, and its perceived importance or popularity in the field [14]. For the unsupervised methods, we explore how different parameters and parameter-value variations influence accuracy. We identify the type of simulated data best suited to assess these methods, and show that properties of the generative network, especially its size, significantly influence prediction accuracies of the methods. We also evaluate these methods using empirical microarray data from normal ovarian tissue. Finally, we compare the best-performing unsupervised methods with the supervised method using simulated datasets obtained from the DREAM3 and DREAM4 competitions [15,29] and datasets generated using the SynTReN software [30]. We selected SynTReN rather than GeneNetWeaver (the simulator used in DREAM3 and DREAM4) because the former is computationally more efficient and allowed us to vary independently the numbers of samples and network nodes.

We measure prediction accuracy by the AUC. Other measures, including sensitivity, specificity, precision, Matthews correlation coefficient and F1 score, have also been used [31-33]. In contrast to AUC, however, these measures require the selection of a threshold that transforms edge weights into interactions and non-interactions, essentially defining a point on the ROC curve. This raises the question of how (at what point on the ROC curve) to define the threshold. Various approaches have been proposed [20,34,35], but since the range and distribution of network weights is method-dependent, a fair comparison of methods is guaranteed only for individually optimized thresholds, for example, maximized F1 score. AUC allows unbiased comparison without the need to optimize a threshold, and has the added advantage of facilitating the comparison of our results with those from DREAM.

Using the best-performing method, we infer normal as well as ovarian cancer GRNs, and seek independent support in the literature and via computational prediction of transcription factor (TF) binding sites (TFBSs). For interactions with a confidently predicted TFBS but without independent literature support, we develop mechanistically detailed case studies that imply novel testable hypotheses of genetic regulation in normal and cancerous ovaries. We perform a topological analysis of the inferred network, revealing a large number of interactions disrupted in cancer and implicating a regulatory switch controlling angiogenesis in ovarian cancer. Finally, we conduct druggability analysis of gene products from high-confidence target genes and angiogenesis-specific genes.

## Materials and methods

### Gene regulatory network inference methods

We selected for comparison eight state-of-the art unsupervised GRNI methods: Relevance Networks (RN) [36], Minimum Redundancy/Maximum Relevance Networks (MRNET) [33], Context Likelihood Relatedness (CLR) [37], The Algorithm for the Reconstruction of Accurate Cellular Networks (ARACNE) [38], Partial Correlation and Information Theory (PCIT) [39], Weighted Gene Co-expression Network Analysis (WGCNA) [40], Gene Network Inference with Ensemble of Trees (GENIE3) [41], and CORRELATIONS [42]. We also worked with one supervised method, Supervised Inference of Regulatory Networks (SIRENE) [43]. All unsupervised methods are implemented in the R language, and SIRENE in MATLAB. For descriptions of the underlying mathematical formalisms, the methods themselves, and the parameters we found useful for optimization, see supplemental material and Table S1 in Additional file 1.

### Datasets

We downloaded simulated knock-down and multifactorial gene expression datasets (each with 100 genes and 100 samples) from the DREAM3 and DREAM4 competitions, along with the associated reference networks [14,15,29]. These data were generated using GeneNetWeaver version 2.0 [29]. The knock-down data contain steady-state expression levels for wild type and for knock-downs of every gene in the network. The multifactorial dataset contains steady-state levels obtained by applying multifactorial perturbations to the original network, and is thought to resemble a real dataset in which each sample is a genome-wide expression profile from a different patient [14,15].

We generated a second set of simulated datasets using SynTReN (Synthetic Transcriptional Regulatory Network) generator version 1.1.3 [30]. It samples from known *Saccharomyces cerevisiae *and *Escherichia coli *networks to create sub-networks, for which it simulates expression data based on Michaelis-Menten and Hill kinetics. Using SynTReN we generated 12 benchmark datasets (3 node numbers × 4 sample numbers) from each of the three topologically different source networks using default parameter settings (Tables S2 and S3 in Additional file 1). The source networks, two from *E. coli *(large and small) and one from *S. cerevisiae*, have different topological properties. Specifically, the networks differ in their numbers of nodes, numbers of interactions, average directed path lengths, and average clustering coefficients. The *E. coli *large network has more nodes and interactions, a longer average directed path length and a higher average clustering coefficient than the *S. cerevisiae *or the *E. coli *small source networks (Table S4 in Additional file 1) [30]. While the sub-networks we extract have identical numbers of nodes, the number of edges varies based on the source network; for example, the 50-node sub-network extracted from *E. coli*-small contains 101 edges, whereas the network of the same size extracted from *E. coli*-large contains 171 edges. For each sub-network, we used SynTReN to simulate multifactorial expression datasets with 10, 50, 100 and 200 samples.

The ovarian cancer microarray dataset (NCBI Gene Expression Omnibus GSE14407) [44] is based on 12 normal surface epithelial cell samples and 12 unmatched cancerous epithelial cell samples isolated by laser-capture microdissection from human serous papillary ovarian adenocarcinoma [44]. We performed data processing and statistical analyses using CARMAweb [45], and 282 differentially expressed genes (*P *< 0.05) identified using SAM [45,46] were input to SIRENE for network inference. In the absence of a reference ovarian GRN, we derived a network from experimentally validated regulatory interactions in TRANSFAC [47] by mapping individual genes from the ovarian cancer dataset onto the reference network, yielding a network of 6,330 interactions among 280 TFs and 2,170 targets (Additional file 2).

To validate our results on the ovarian cancer dataset described above, we also applied SIRENE to a dataset by Tothill *et al. *[48] downloaded from NCBI Gene Expression Omnibus (GSE9891). This dataset was likewise created on the Affymetrix HG_U133_plus2 platform and is composed of 285 patient samples. This dataset does not contain data from normal ovary tissue. We selected patient samples with serous adenocarcinoma stage 3 with grade 2 or 3, resulting in a reduced dataset with 158 patients (98 grade 3 and 60 grade 2). We obtained the expression profiles for the 282 differentially expressed genes from the 158 patients selected, and employed SIRENE to infer the regulatory network for this dataset.

### Evaluation

To measure prediction accuracy against a corresponding reference network, we used the AUC [20], a single measure that summarizes the trade-off between true positive rate and false positive rate [20]. An AUC value of 0.5 corresponds to a random prediction, while a value of 1 indicates perfect prediction.

To investigate whether evidence for interactions exists in the literature, we queried GeneGO [49], Ingenuity Pathway Analysis [50] and PubMed abstracts, the latter via PubGene (now Coremine) [51]. For GeneGO and IPA, we uploaded the set of target genes as a list, retrieved all regulatory interactions without restricting the search, and looked for regulatory interactions identified in our predicted network. For PubGene, we queried with predicted TF-target gene pairs, searching across human and other species.

For each predicted regulatory interaction (TF-target gene pair) we applied Genomatix MatInspector [52] to determine whether a TFBS for that TF is present upstream of the target gene. For each TFBS match, this algorithm assigns a matrix similarity score ranging from 0 to 1 (exact match). We queried MatInspector using Entrez Gene Identifiers and a promoter sequence length 2,000 bp upstream of the transcriptional start site.

Functional enrichment analysis of gene lists was performed using the DAVID webtool [53,54]. For any Gene Ontology (GO) term, a modified Fisher exact test was applied to determine whether the number of genes annotated with a particular GO term is enriched in the gene list compared to the number with that GO term in the background. We set the HG-U133 Plus 2.0 array, as well as genes present in the network, as background.

### Network inference

To generate the normal and the cancer GRNs, the 282 differentially expressed genes and associated reference TF-target networks with 115 interactions, between 9 TF and 106 target genes, obtained from TRANSFAC were input to SIRENE. Parameters used for network generation are provided as Table S5 in Additional file 1. The resulting networks were visualized and analyzed using Cytoscape 2.8 [55]. Network interactions were rendered according to evidence.

### Druggability analysis

Druggability analysis of 178 proteins encoded by all genes in the predicted ovarian network (above) was conducted using the CancerResource [56] and PharmGKB [57] webtools and databases.

## Results

### Comparative evaluation

#### Parameter settings affect accuracy of GRNI methods

Most of the eight unsupervised methods evaluated here can be tuned by selection of parameter values. To study the effect of parameter variation on performance, and to optimize parameter values, we used the DREAM4 multifactorial simulated expression data [29].

**Figure 1:**
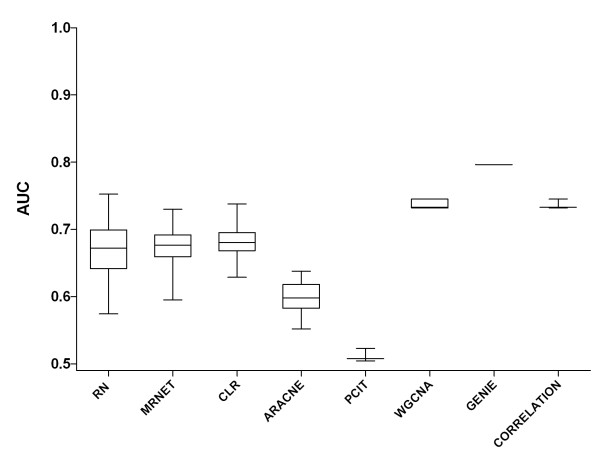
**Parameter optimization of methods**. Comparison of unsupervised GRNI (gene regulatory network inference) methods using the DREAM4 multifactorial dataset. Each boxplot represents variation in prediction accuracy over the different parameter values used for optimization. With GENIE (Gene Network Inference with Ensemble of Trees), no parameter was found useful for optimization, so it was used with default settings. For information on the complete parameter sweep see Figure S1 in Additional file 3.

Figure 1 shows, for each method, the range of prediction accuracies we observed by varying parameter values. For the mutual information (MI)-based methods (RN, MRNET, CLR and ARACNE) we examined three parameters: MI estimators, discretization methods and bin size. We optimized four different MI estimators (mi.empirical, mi.mm, mi.shrink, and mi.sg) and three discretization methods (equal frequency, equal width, and global equal width). For each discretization method we furthermore varied the bin number between 2 and 95 (from 2 to 10 with increment 1, and thereafter with increment 5). Thus, in total, for each method we examined 312 parameter values (4 MI estimators × 3 data discretization methods × 26 bin sizes). For PCIT, WGCNA and CORRELATIONS we evaluated three correlation methods: Pearson, Spearman and Kendall-Tau. In addition, for WGCNA we varied the softpower parameter [40] between 7 and 17. For each of PCIT, CORRELATIONS and WGCNA we examined 3, 3, and 33 parameter values, respectively. RN showed the largest variation in prediction accuracy and WGCNA and CORRELATIONS showed the least. GENIE achieved the best prediction accuracy on these data, as it did in DREAM4 [41]. We found that bin numbers between 3 and 6 gave the best performance irrespective of the combination of GRNI, MI estimator and discretization method (Figure S1 in Additional file 3). To examine the robustness of parameter optimization, we repeated the optimization process on other datasets (Table S6 in Additional file 1) and found that the optimal parameter values changed with different datasets, that is, there is no 'one size fits all' set of parameter values.

#### Data type is critical for performance of all GRNI methods

**Figure 2:**
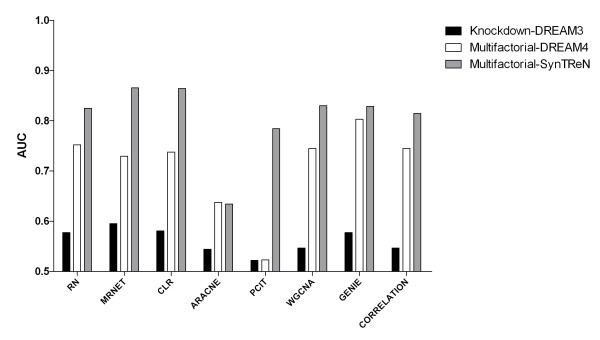
**Accuracies of gene regulatory network inference methods on two different data types**. Comparison of unsupervised GRNI methods on two different data types, knockdown, and multifactorial with 100 genes and 100 samples.

To investigate the influence of data type on performance, and to identify the most-informative type of simulated data, we tested all methods on two different DREAM data types (knock-down and multifactorial [29]) and on multifactorial data generated using SynTReN (Figure 2). All methods were run using optimal parameter settings obtained for the respective dataset. We found the prediction accuracies of all methods extremely low on the knock-down data, implying that these data are less informative, and reasonably high (AUCs around 0.8 for most methods) on the multifactorial data. ARACNE achieved low accuracies in general and PCIT worked only well for SynTReN data. Between the two multifactorial datasets, accuracies are generally higher on the SynTReN data than DREAM, suggesting that not only the experimental type but also the process of simulation can affect performance.

#### Network properties influence accuracy

**Figure 3:**
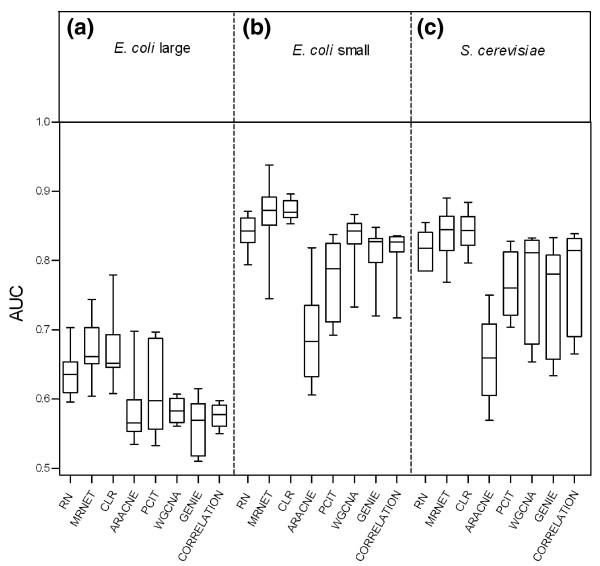
**Accuracies of gene regulatory network inference methods on different networks**. **(a-c) **Comparison of accuracies (AUCs) of unsupervised GRNI methods on the sub-networks extracted from three source networks: *E. coli *large (a), *E. coli *small (b), and *S. cerevisiae *(c). Each boxplot represents variation in the accuracy of that method obtained using optimal parameter settings for each of the 12 datasets generated by SynTReN. The highest accuracies were achieved on the small *E. coli *networks.

Because network properties, including number of nodes, edges and network motifs, may influence the performance of GRNI methods [13,15,29], we evaluated each method against sub-networks of sizes 50, 100 and 200 nodes generated from three source networks using SynTReN (Materials and methods), and using optimized parameter value settings for each method. Figure 3 shows, for each GRNI method, the range of prediction accuracies achieved. We observed that the median accuracies of all methods are significantly higher on sub-networks extracted from the *E. coli*-small and *S. cerevisiae *source networks than on the *E. coli*-large networks (Mann-Whitney U-test, *P *< 0.0003 with Bonferroni correction, significance threshold α = 0.01). Accuracies do not differ significantly on the *E. coli*-small and *S. cerevisiae *networks (Mann-Whitney U-test, *P *> 0.0003 with Bonferroni correction, α = 0.01) (Table S7 in Additional file 1). For comparison of prediction accuracies on individual datasets, see Figure S2 in Additional file 4. The consistently lower accuracies for the large *E. coli *network in comparison to *E. coli*-small may result from the existence of more-complicated regulatory motifs and the higher edge density within the former [31,58].

#### Performance of unsupervised GRNI methods on empirical data

**Figure 4:**
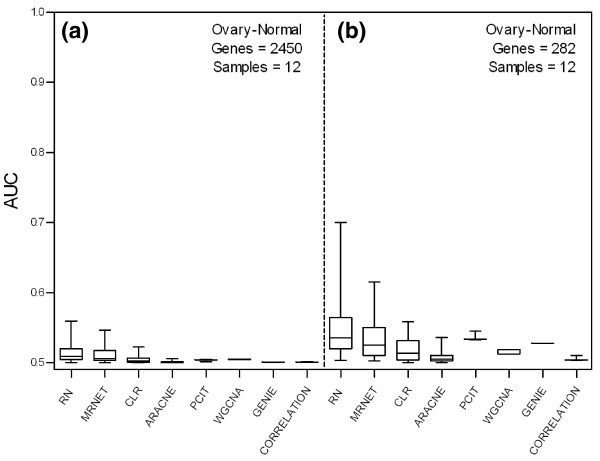
**Accuracies of gene regulatory network inference methods on empirical data**. Accuracies (AUCs) of unsupervised GRNI methods on normal ovarian microarray data. **(a) **Prediction accuracy of methods on normal ovarian data with 2,450 genes and 12 samples. **(b) **Prediction accuracy of methods on normal ovarian data with 282 differentially expressed genes and 12 samples.

To assess the performance of GRNI methods on real datasets and evaluate their potential in analyzing cancer expression data, we examined their application to two subsets of an ovarian microarray dataset [44] with 12 samples and 2,450 genes (Figure 4a) and 282 genes (Figure 4b), respectively. We found prediction accuracies of all the methods to be extremely low on these datasets, particularly on the larger dataset, most likely due to the very small number of samples in relation to genes. Only RN and MRNET showed some predictive power on the smaller dataset for optimal parameter settings.

#### Comparison of best unsupervised methods with a supervised method

Table 1 compares prediction accuracies of unsupervised and supervised GRNI methods on simulated and empirical data. The best-performing unsupervised method for each dataset is compared with the supervised method SIRENE. We found that SIRENE always performs better than the best-performing unsupervised method except on the DREAM4 dataset. The highest accuracy of SIRENE is seen when the method is applied to the small normal ovarian dataset (AUC = 0.86).

**Table 1:** Accuracies of unsupervised and supervised GRNI methods on different datasets

	Unsupervised method		SIRENE
		
Datasets	Method	AUC	AUC
DREAM3 (knockdown): genes 100, samples 100	MRNET	0.59	0.71
DREAM4 (multifactorial): genes 100, samples 100	GENIE	0.79	0.69
Ovary-normal: genes 2,450, samples 12	RN	0.55	0.62
Ovary-normal: genes 282, samples 12	RN	0.70	0.86

Comparison of accuracies (AUC) of unsupervised and supervised Gene Regulatory Network Inference (GRNI) methods on different datasets. For each dataset, the best-performing unsupervised method was selected for comparison with SIRENE.

AUC, area under the receiver-operating characteristic curve; DREAM, Dialogue for Reverse Engineering Assessments and Methods; GENIE, Gene Network Inference with Ensemble of Trees; MRNET, Minimum Redundancy/Maximum Relevance Networks; RN, Relevance Networks; SIRENE, Supervised Inference of Regulatory Networks.

### Application of GRN inference to ovarian cancer data

The above evaluation gives us some confidence that GRNI methods can predict small GRNs (Figures 1 to 3). We now apply the best-performing method, SIRENE, to ovarian cancer data with 282 differentially expressed genes and predict GRNs for normal and cancerous ovarian epithelial tissue. We evaluate all predicted interactions, as well as the network itself, to determine if GRNI yields novel insights.

#### Structural variation between normal and cancer networks

**Figure 5:**
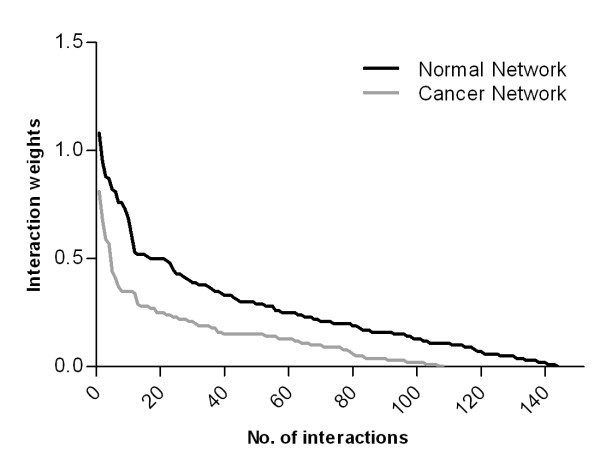
**Structural variation between the normal and cancer networks**. Comparison of interaction weights predicted by SIRENE for normal and cancer.

**Figure 6:**
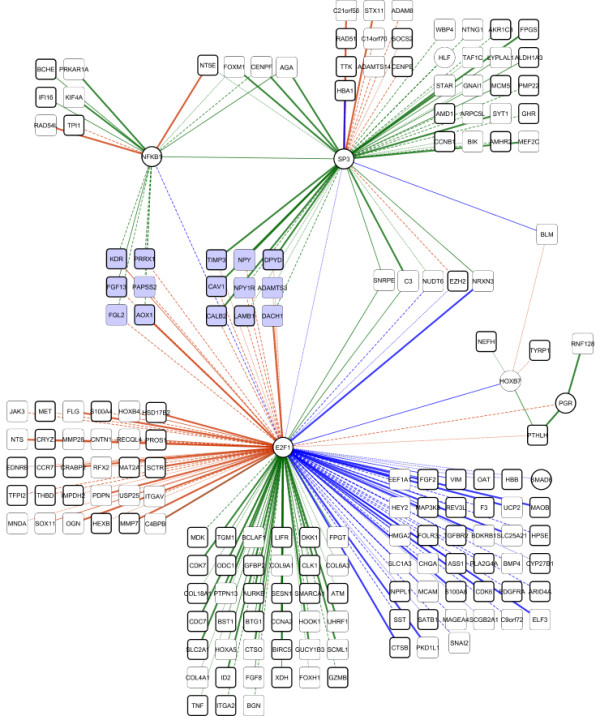
**The ovarian gene regulatory network**. The ovarian network inferred using SIRENE, showing target genes (rectangles) and transcription factors (circles). Two clusters of genes (shaded blue, in the centre of the figure) switch regulators between the two conditions, controlled by SP3 or NFκB1 in normal and by E2F1 in cancer. Bold nodes are known to have protein products that are targeted by anti-cancer drugs. Edge colors: green, normal; orange, cancer; blue, both. Edge line type: bold, literature and TFBS; solid, literature; dashed, TFBS; dotted, no evidence.

Figures 5 and 6 show structural variation between the normal and the cancer GRNs inferred using SIRENE (the full networks are provided as Additional files 5 and 6). SIRENE assigns positive weights to indicate interactions, and negative weights to indicate absence of interactions, while the absolute weight reflects the confidence in the prediction. From Figure 5, we see that more interactions (144) are predicted in the normal than in the cancer network (108), and that the interaction weights are larger in the former. In total, SIRENE predicted 205 interactions, 97 specific to normal, 61 specific to cancer, and 47 present in both networks (Figure 6).

#### Literature validation and computational prediction of TF binding sites

We next asked whether any of these 205 predicted interactions had previously been reported in the literature. Using PubGene and the manually curated GeneGo and IPA data sources, we found prior evidence for 93 of our predicted interactions (Additional file 7). Promoter analysis of the 205 target genes using Genomatix MatInspector revealed upstream TFBSs for 124 interactions (Additional file 6), 67 of which had no previous literature evidence. Combining these results, we see that 78% of the interactions predicted by SIRENE have supporting evidence either from literature or from TFBS prediction. These results are only slightly lower than the accuracy rate calculated for SIRENE on the normal ovarian data (as assessed using an independent reference network), and slightly higher than expected based on the performance of SIRENE on the synthetic DREAM datasets.

#### Prediction of novel interactions

We examined in detail the ten interactions most confidently (weight ≥ 0.5) predicted interactions confidently predicted by SIRENE but not reported in the literature (Table S8 in Additional file 1). For seven of these we predict a high-quality TFBS; below, for two of these, we propose a role and mechanism of action in normal ovaries, or in ovarian cancer.

#### E2F1 and DKK1

E2F1 is a member of the E2F family of transcription factors best known for regulating cell-cycle progression. In ovarian cancer, up-regulation of E2F1 contributes to uncontrolled cell proliferation. E2F1 is regulated by the WNT/β-catenin/Tcf pathway [59]. Importantly, E2F1 itself can repress the WNT pathway by direct up-regulation of proteins such as AXIN1/2 and SIAH1, indicating the presence of a feedback loop between E2F-1 and the WNT pathway [60,61]. WNT signaling plays important roles in development, differentiation and cell proliferation, and activated WNT signaling has been implicated in a wide range of cancers [62]. DKK-1 is a secreted glycoprotein that acts as a specific antagonist of WNT signaling; up-regulation of this pathway due to down-regulation of DKK-1 has been implicated in several cancers, and inhibition of WNT signaling by DKK-1 inhibits ovarian carcinoma cell proliferation [63].

Our GRNI analysis suggests that E2F1 interacts with DKK-1 in the normal ovary, but that this interaction is lost in ovarian cancer. Furthermore, our analysis of the ovarian cancer data indicates that E2F1, and direct downstream targets of the WNT pathway (survivin, ID2 and vimentin) critical in cell-cycle progression, are up-regulated. We hypothesize that in normal ovarian epithelia, E2F1 turns on expression of DKK-1, which in turn inhibits the WNT pathway, which ultimately stops the expression of cell-cycle genes, whereas in ovarian cancer, disruption of E2F1 regulation of DKK1 results in loss of DKK1 expression and corresponding activation of the WNT pathway, ultimately resulting in activation of cell-cycle genes.

#### E2F1 and HSD17B2

Increased estrogen levels contribute to the risk of ovarian cancer, but the exact mechanism is poorly understood. Estradiol is the most potent biologically active form of estrogen in ovarian tissue. Estradiol is mitogenic, and evidence suggests that an increase in intra-tumoral estradiol may play a causative role in tumorigenesis [64]. 17β-Hydroxysteroid dehydrogenase type 2 (HSD17B2), a member of a family of enzymes that regulate intra-tissue estrogen synthesis by catalyzing the interconversion of estradiol with the weakly estrogenic estrone, is critical for normal endometrial growth and differentiation [64]. The inactivation of estradiol by HSD17B2 protects against over-proliferation in estradiol-responsive tissues. The mechanism of regulation for this enzyme is not known. Previous studies have shown abnormally elevated levels of estradiol in cancer tissue, causing cell proliferation and tumor growth [65].

In addition to the involvement of E2F1 in WNT signaling, E2F1 is also involved in the estrogen-triggered regulation of cell proliferation [66]. E2F1 is a direct target of ESR1, which promotes cell proliferation through E2F1 target genes. Knock-down of E2F1 blocks estrogen regulation of E2F1 target genes, implying that E2F1 is critical for estrogen-regulated proliferation of cancer cells [66]. We see that E2F1 expression is elevated in ovarian cancer, while HSD17B2 expression is reduced. Thus, we predict that E2F1 negatively regulates HSD17B2 in ovarian cancer and that reduced HSD17B2 results in an excess of estradiol, which in turn activates cell-proliferation genes through the activation of ESR1.

#### The predicted ovarian gene regulatory network

The ovarian network, including regulatory interactions predicted for both normal and cancerous ovarian data, is presented in Figure 6. This network includes seven TFs and 171 TF-target genes. Judged by number of connections, by far the most influential TF in the network is E2F1, which interacts with 134 other genes, including five of the remaining six TFs. Two other TFs, SP3 (51 targets) and NFκB1 (18 targets), also engage in many regulatory interactions, while the remaining TFs (HOXB7, PGR, SMAD6 and HLF) together account for only 10 regulatory interactions.

Topological analysis of the network reveals a set of 15 target genes that are regulated by SP3 or NFκB1 in normal cells, but by E2F1 in ovarian cancer (Figure 6). GO enrichment analysis, using these 15 target genes against the HG-U133 Plus 2.0 array gene sets as a background in DAVID, revealed angiogenesis as a broad enrichment for the nine SP3 targets, and mesenchymal cell proliferation for the six NFκB1 targets. As mesenchymal cell proliferation is involved in angiogenesis [67], this set of 15 genes (Table S9 in Additional file 1) constitutes an angiogenic sub-network, or program, whose transcriptional regulation is dramatically altered in ovarian cancer. The full results of the enrichment analysis are presented in Additional file 8. E2F1, SP3 and NFκB1 have well-documented roles in angiogenesis [68-70].

Neither angiogenesis nor the transcription factors E2F1, SP3 and NFκB1 were identified in the original analysis of the ovarian cancer data [44]. The specific role(s) of these TFs in ovarian cancer is poorly understood, and we find no reports implicating a switch in regulation of angiogenesis in ovarian cancer. These results highlight the novel insights and hypotheses that can result from application of GRNI to cancer microarray data.

#### Validation on an independent dataset

To validate the results achieved on the ovarian cancer dataset, we also employed SIRENE to infer a GRN from a second, larger (158 sample) dataset derived from a dataset used by Tothill *et al. *[48]. The inferred ovarian cancer GRN is provided as Additional file 9. Edge overlap analysis between this GRN and the ovarian cancer GRN inferred previously (above) shows 64% edge overlap overall, and 85% edge overlap for the 20 interactions predicted with highest confidence. This level of agreement strongly indicates that most of the interactions were reliably identified.

#### Druggability analysis of protein products of target genes

We conducted druggability analysis of the proteins corresponding to genes in our predicted ovarian GRN using CancerResource [56], a comprehensive knowledgebase of experimentally validated drug-target relationships. To identify the proteins regarded as anti-cancer drug targets, we input all 178 proteins from our GRN to CancerResource. We find that 61% of the proteins from our network are targeted by at least one anticancer drug (Figure 6; Additional file 10). In many cases a single drug targets multiple proteins, or conversely multiple drugs target a single protein (Additional file 10). Here we present the results for 24 genes: 10 genes involved in the interactions most confidently predicted (weight ≥0.5) by SIRENE (Table S8 in Additional file 1), and 15 angiogenesis-specific genes (described above) that are differentially regulated in normal ovary and ovarian cancer (Figure 6). One gene, *NPY1R*, is common to both sets. Table 2 shows drugs identified as targeting the protein products of these genes. Of the 24 gene products, 16 are targeted by anti-cancer drugs. Two additional genes (*NPY *and *NPY1R*) produce products targeted by other classes of drugs (selective serotonin reuptake inhibitors and selective beta-2-adrenoreceptor agonists). Overall, our analysis indicates that 18 of these 24 proteins can be targeted by approved (including experimentally approved) drugs. Products of six genes (AGA, NTNG1, ADAMTS3, DACH1, FGL2 and PAPSS2) are not known to be drug targets.

**Table 2:** Druggability analysis results

Gene name	Gene type	Targeted drugs
**Top 10 target genes**		
*BCHE*	Enzyme	Bicalutamide, genistein, choline, isoflurophate, hexafluorenium, demecarium bromide, echothiophate iodide, butyric acid
*CDK7*	Protein kinase	Lycopene, genistein, flavopiridol
*DKK1*	Receptor ligand	Decitabine
*CCR7*	GPCR	Decitabine
*TPI1*	Enzyme	Fluorouracil, quercetin
*HSD17B2*	Enzyme	NADH
*HBB*	Transporter	Iron-dextran complex
**Angiogenesis genes: SP3 targets**		
*TIMP3*	Binding protein	Salinomycin, decitabine, sulindac, adaphostin
*CAV1*	Binding protein	Decitabine, progesterone, mifepristone
*CALB2*	Binding protein	Oxaliplatin, fluorouracil
*LAMB1*	Receptor ligand	Benzamidine, carebastine, anistreplase, tenecteplase
*DPYD*	Enzyme	Oxaliplatin, gemcitabine, docetaxel, s1(combination), capecitabine, cisplatin, fluorouracil, tegafur, carboplatin, paclitaxel, genistein, enfuvirtide, raltitrexed, amifostine, irinotecan, methotrexate, mitoguazone, uracil
**Angiogenesis genes: NFκB1 targets**		
*KDR*	Receptor with enzyme activity	Epigallocatechin gallate, resveratrol, sorafenib, sunitinib, bevacizumab, sirolimus, conivaptan, zonampanel, SU6668, vatalanib, vandetanib, axitinib, cediranib, trapoxin, motesanib, E-7080, erlotinib, Ca0456456, geldanamycin
*FGF13*	Receptor ligand	Bicalutamide
*PRRX1*	Transcription factor	Alitretinoin
*AOX1*	Enzyme	Isovanillin, norcantharidin, NSC336628

Genes and anti-cancer drugs targeting their products were obtained using Cancer Resource and PharmGKB webtools and databases. GPCR, G-protein-coupled receptor.

## Discussion

In this study we have undertaken a comparative evaluation of the performance of eight unsupervised and one supervised methods of GRNI, using synthetic and empirical cancer datasets. How reliably these methods perform on real data is a vital consideration for cancer researchers. Our application of the best-performing method, SIRENE, to real ovarian cancer data demonstrates that GRNI can be reliable (as evidenced by experimentally based literature not used in our inference) and predict novel interactions that are biologically and mechanistically reasonable (hence worthy of prioritization for laboratory-based experimental validation).

Parameter settings are crucial for optimal performance of GRNI methods, and indeed we usually observe large variations in accuracy when parameter values are changed. While parameter-value optimization can be time-consuming, we strongly recommend it as part of computational protocols including GRNI.

We observe higher accuracies on simulated multifactorial than on knock-down data. As the former are considered to resemble empirical gene-expression data more closely than do other types of synthetic data, this gives reason for optimism that GRNI methods can usefully be applied to clinical data. The evaluation of GRNI methods on real data is difficult, since a true reference network is usually lacking. Here we used TRANSFAC to estimate the true transcriptional network for ovarian data; even so, the TRANSFAC-based network is likely to contain interactions not present in ovarian epithelium, and potentially misses ovary-specific regulatory interactions. Integrating available networks with tissue-specific transcriptional interactions generated using techniques like ChIP-seq or ChIP-chip has the potential to improve training and evaluation of GRNI methods on real data in the near future.

In agreement with others [14,31,71], we find that GRNI methods are typically more accurate on simulated than on real data. This may be due in part to topological or other mismatch with the reference network (above), but the presence of multilayered direct and indirect regulatory controls, including chromatin remodeling, microRNAs and metabolite-based feedback in a real GRN [3], is likely to make the network inference problem more challenging.

In agreement with other studies [8], we found SIRENE to be a more accurate predictor than the unsupervised methods evaluated (Table 1), presumably because supervised methods take advantage of known regulatory data in the training process. One of the major difficulties in adopting supervised methods has been the lack of a true or known network. Here we trained on a network of regulatory interactions extracted from TRANSFAC; others have used regulation data from RegulonDB [43] or KEGG (Kyoto Encyclopedia of Genes and Genomes) [72]. Nonetheless, such approaches do not capture a true tissue-specific GRN, which, if available, would probably further improve the accuracy of supervised methods on large-scale data.

Topological analysis of the combined networks revealed that many predicted interactions are disrupted in cancer, with E2F1, SP3 and NFκB1 emerging as major regulators (Figure 6). Interestingly, we predict that the hormone-responsive TF progesterone receptor plays only a minor role in the regulation of differentially expressed genes. Annotating nodes for druggability adds an additional dimension to the interpretation of the network, specifically identifying TFs (that is, E2F1, SP3, NFκB1, PGR and SMAD6) that can be targeted by approved anti-cancer drugs, presenting the possibility for intervening pharmaceutically to change the activity of these regulatory sub-networks.

Topological analysis of the complete network also suggests cross-regulation of angiogenesis-specific genes through SP3, NFκB1 and E2F1 in the normal and ovarian cancer networks, and we hypothesize that deregulation of these angiogenic genes may be associated with oncogenesis. Indeed, key interactions in this sub-network include the regulation of KDR and VIM by E2F1. KDR is a key player in initiating angiogenesis and a drug target in several cancers, including ovarian carcinoma [73], while VIM is a marker of the epithelial-mesenchymal transition, and there is growing evidence of its involvement in epithelial cancers [74].

Based on our structured survey of published literature, we propose functional models for two potential novel interactions: E2F1 with DKK1 via WNT signaling, and E2F1 with HSD17B2 via estrogen synthesis. Independent of our analysis, there is evidence supporting the presence of an E2F1-binding site in the DKK1 promoter [75], which further supports our prediction. This illustrates the ability of GRNI to reveal interactions that have not yet been validated.

## Conclusions

Our study represents a concrete application of GRNI to ovarian cancer, demonstrating how this approach can discover novel gene regulatory interactions and uncover deregulation of critical processes, such as angiogenesis, which otherwise may not be detected by classical microarray data analysis. We present the complete cycle of computational systems biological research, from genome-scale data analysis via GRNI and evaluation of methods, to prediction of novel, testable hypotheses and generation of new insight. Especially when integrated with experimental validation, GRNI can be a powerful tool in understanding how regulatory networks are disrupted and rewired, identifying novel regulatory interactions as well as broader systemic disruptions in key oncogenic processes.

## Abbreviations

ARACNE: The Algorithm for the Reconstruction of Accurate Cellular Networks; AUC: area under the receiver-operating characteristic curve; bp: base pair; CLR: Context Likelihood Relatedness; DREAM: Dialogue for Reverse Engineering Assessments and Methods; GENIE: Gene Network Inference with Ensemble of Trees; GO: Gene Ontology; GRN: gene regulatory network; GRNI: gene regulatory network inference; MI: mutual information; MRNET: Minimum Redundancy/Maximum Relevance Networks; PCIT: Partial Correlation and Information Theory; RN: Relevance Networks; ROC: receiver-operating characteristic; SIRENE: Supervised Inference of Regulatory Networks; TF: transcription factor; TFBS: transcription factor binding site; WGCNA: Weighted Gene Co-expression Network Analysis.

## Competing interests

The authors declare that they have no competing interests.

## Authors' contributions

PBM carried out all experiments and analyzed the data. SRM assisted with comparative evaluation workflow and technical interpretation. MJD assisted with biological interpretations and figure generation. AR assisted with network inference methods set up. MAR assisted with experiment design and supervised the project. PBM, SRM, MJD and MAR contributed to writing the manuscript. All authors read and approved the final manuscript.

## Supplementary Material

Additional file 1 **Supplemental methods and results**. Supplemental methods and results [76-91]. Click here for file

Additional file 2 **TRANSFAC network**. Reference network derived from TRANSFAC database for ovarian cancer microarray data. Click here for file

Additional file 3 **Figure S1 - prediction accuracies of MI-based methods on the multifactorial DREAM4 data**. Prediction accuracies of MI based methods on the multifactorial DREAM4 data for all the parameter values investigated. Click here for file

Additional file 4 **Figure S2 - prediction accuracies of methods on datasets generated from three different source networks**. Prediction accuracies of methods on 12 different datasets generated from three different source networks: *E. coli *large, *S. cerevisiae *and *E. coli *small. Click here for file

Additional file 5 **Normal and ovarian cancer network**. The normal and cancerous ovarian GRN inferred using SIRENE. Click here for file

Additional file 6 **Visualization of ovarian network**. Cytoscape file for the network visualization. Nodes and edges loaded with attributes. Click here for file

Additional file 7 **Literature and TFBS evidence for predicted interactions**. Validations of predicted interactions in normal and ovarian cancer network inferred using SIRENE. Click here for file

Additional file 8 **DAVID enrichment analysis results**. Functional enrichment analysis results of angiogenesis-specific genes using DAVID. Click here for file

Additional file 9 **Ovarian cancer GRN inferred using a second, independent dataset**. Ovarian cancer GRN inferred using SIRENE. Expression data from 158 serous ovarian adenocarcinoma patients were extracted from Tothill *et al. *[48]. Click here for file

Additional file 10 **Druggability analysis results**. Genes and anti-cancer drugs interaction matrix obtained from the druggability analysis of all target genes in the ovarian GRN. The analysis was conducted using CancerResource webtool and database. Click here for file
