# Supplementary material for: Gene regulatory network inference: evaluation and application to ovarian cancer allows the prioritization of drug targets
Source: Genome Med. 2012 May 1;4(5):41. doi: 10.1186/gm340 (PMC3506907; doi:10.1186/gm340)
Supplement: Additional file 1 — Supplemental methods and results. Supplemental methods and results [76-91]. [file gm340-S1.DOC]

# Supplemental material to:

**Gene regulatory network inference: evaluation and application to ovarian cancer allows the prioritization of drug targets**

Piyush B. Madhamshettiwar^1^, Stefan R. Maetschke^1^, Melissa J. Davis^1,2^, Antonio Reverter^3^ and Mark A. Ragan ^1§^

^1^The University of Queensland, Institute for Molecular Bioscience, 306 Carmody Road, St. Lucia, Brisbane, Queensland 4072, Australia

^2^Queensland Facility for Advanced Bioinformatics, 306 Carmody Road, Brisbane, Queensland 4072, Australia

^3^CSIRO Livestock Industries, 306 Carmody Road, St. Lucia, Brisbane, Queensland 4072, Australia

^§^Corresponding author

Email addresses:

PBM: p.madhamshettiwar@imb.uq.edu.au

SRM: s.maetschke@uq.edu.au

MJD: m.davis@imb.uq.edu.au

AR: toni.reverter-gomez@csiro.au

MAR: m.ragan@uq.edu.au

**Supplemental Materials and Methods**

## Network inference methods

In this section we provide a more detailed description of the network inference methods evaluated in this study.

***Correlation***

Correlation-driven network inference methods are based on the assumption that correlated expression levels between two genes are indicative for a regulatory interaction. Correlation coefficients range from +1 to -1 and a positive correlation coefficient indicates an activating interaction, while a negative coefficient indicates an inhibitory interaction. The most commonly used correlation measure is the one by Pearson and is defined as

$corr(X_{i},X_{j})=\frac{\mathrm{cov}(X_{i},X_{j})}{\sigma(X_{i})\sigma(X_{j})}$,

where *X_i_* and *X_j_* are the expression levels of genes *i* and *j*, *cov(⋅,⋅)* denotes the covariance between two expression profiles and σ(⋅) is the standard deviation. Person's correlation measure assumes normally distributed values, an assumption that does not necessarily hold for gene expression data. Therefore rank-based measures are frequently employed, with the measures by Spearman and Kendall being the most common ones. Spearman's method is simply Pearson's correlation coefficient for the ranked expression values, and Kendall's tau(τ) coefficient is computed as

,

where *X_i_* and *X_j_* are the ranked expression levels of genes *i* and *j*. *con(⋅,⋅)* denotes the number of concordant and *dis(⋅,⋅)* the number of disconcordant value pairs in *X_i_* and *X_j_*, where both variables contain *n* values.

Note that correlation measures are symmetric and therefore undirected. To assign directionally to inferred interactions additional information, e.g. which genes are acting as transcription factors, is required.

***WGCNA (Weighted Gene Co-expression Network Analysis)***

WGCNA is a simple modification of the correlation based inference method described above that emphasizes high correlation values by raising the absolute value of the coefficient to the power of β ≥ 1 (softpower). More formally, the weight *w_ij_* of an interaction between two genes *i* and *j* is computed as

$$w_{ij}=\left| corr(X_{i},X_{j}) \right|^{\beta} .$$

***RN (Relevance Network)***

The relevance network approach is similar to the correlation-driven network inference methods discussed before but measures mutual information (MI) between gene expression profiles instead of correlation to predict interactions. The mutual information *I* between two, discrete variables *X_i_* and *X_j_* is defined as

$I(X_{i},X_{j})=\sum_{x_{i}\in X_{i}} \sum_{x_{j}\in Xj} p(x_{i},x_{j})\log\left(\frac{p(x_{i},x_{j})}{p(x_{i})p(x_{j})}\right)$,

where *p(x_i_, x_j_)* is the joint probability distribution of *X_i_* and *X_j_*, and *p(x_i_)* and *p(x_j_)* are the marginal probabilities. *X_i_* and *X_j_* are required to be discrete variables, which in the case of expression data require a discretisation step before the mutual information can be computed. In its simplest form expression values are binned, e.g. up, neutral, down to convert real-valued data to discrete values. More advanced discretisation methods and estimators for mutual information that are available for RN are described in .

Mutual information has the advantage over the correlation-based methods to be able to identify non-linear relationships between variables but does not inform about the type of interaction such as activating or inhibitory. Note that neither correlation-based nor mutual-information-based methods can natively infer the direction of an interaction.

***CLR (context likelihood of relatedness)***

CLR extends the relevance network method (RN) by taking the background distribution of the mutual information values *I(X_i_, X_j_)* into account. The most probable interactions are those that deviate most from the background distribution and for each gene *i* a maximum *z*-score *z_i_* is calculated as

$$z_{i}=\max_{j} \left(0,\frac{I(X_{i},X_{j})-\mu_{i}}{\sigma_{i}}\right) ,$$

where *µ_i_* and *σ_i_* and are the mean value and standard deviation of the mutual information values *I(X_i_, X_k_), k=1,...,n*, respectively. The interaction score *z_ij_* between two genes *i* and *j* is then defined as

$$z_{ij}=\sqrt{z_{i}^{2}+z_{j}^{2}} .$$

The background correction step described above aims to reduce the prediction of false interactions based on false correlations and indirect interactions.

***ARACNE (Algorithm for the Reconstruction of Accurate Cellular Networks)***

ARACNE is another extension of the relevance network (RN) that applies the Data Processing Inequality (DPI) to filter out indirect interactions. DPI states that, if gene X_1_ interacts with gene X_2_ via gene X_3_ then the following inequality holds:

$$I(X_{1},X_{2})\leq\min\left(I(X_{1},X_{2}),I(X_{2},X_{3})\right)$$

ARACNE considers all possible triplets of genes (interaction triangles) and computes the mutual information values for each gene pair within the triplet. Interactions within an interaction triangle are assumed to be indirect and therefore pruned if they violate the DPI by a specified tolerance threshold.

***PCIT (Partial Correlation and Information Theory)***

Similar to ARACNE the PCIT method extracts all possible interaction triangles and applies the DPI to filter indirect interactions but uses partial correlation coefficients instead of mutual information as interaction weights. The partial correlation coefficient *corr_ij_* between two genes *i* and *j* within an interaction triangle *(i,j,k)* is defined as

$$corr_{ij}(X_{i},X_{j},X_{k})=\frac{corr(X_{i},X_{j})-corr(X_{i},X_{k})corr(X_{j},X_{k})}{\sqrt{\left(1-corr(X_{i},X_{k})\right)^{2}\left(1-corr(X_{j},X_{k})\right)^{2}}} ,$$

where *corr(⋅,⋅)* is the Person correlation coefficient. The partial correlation coefficient aims to eliminate the effect of the third gene *k* on the correlation between genes *i* and *j*.

***MRNET (Minimum Redundancy/Maximum Relevance Network)***

The MRNET method employs mutual information between expression profiles and a feature selection procedure (MRMR) to infer interactions between genes. More precisely, the method iterates over all genes, assuming for each gene that it functions as a target gene and all other genes are its regulators. The mutual information values for the target gene and the regulators are computed and MRMR is applied to find the best subset of regulators. MRMR stands for minimum-redundancy-maximum-relevance and selects the genes X_i_^MRMR^ with the largest mutual information value and the smallest redundancy base on the following definition

$$X_{i}^{MRMR}=\arg\max_{X_{i}\in V\backslash S} (u_{i}-r_{i}) ,$$

where Y is the target, and S is the set of selected genes. $u_{i}=I(X_{i},Y)$ is the relevance term (mutual information) and r_i_ is the redundancy term, which is defined as

$$r_{i}=\frac{1}{\left|S\right|}\sum_{X_{k}\in S} I(X_{i},X_{k}) .$$

***GENIE3 (GEne Network Inference with Ensemble of trees)***

GENIE3 is similar to MRNET in that it also lets each gene take on the role of a target regulated by the remaining genes and then employs a feature selection procedure to identify the best subset of regulator genes and therefore interactions. In contrast to MRNET, however, not mutual information and MRMR but Random Forests and Extra-Trees are used for regression and feature selection. GENIE makes few assumptions about the nature of the relationships between the genes and specifically allows the presence of feedback loops in the network.

***SIRENE (Supervised Inference of Regulatory Networks)***

In contrast to the methods described above SIRENE is a supervised method that requires knowledge of interactions and non-interactions between genes, and also a list of regulator genes (transcription factors). For each regulator gene SIRENE trains a binary classifier – a Support Vector Machine with a radial basis function kernel – to discriminate between targets and non-targets of the regulator gene based on their gene expression values. As a consequence of this design, the network is directed and only interactions between transcription factors and non-transcription factors are inferred, but not between transcription factors or between target genes. The method assigns large positive weights to likely interactions and large negative weights to unlikely interactions but does not distinguish between inhibitory or activating interactions.

## Network inference: parameter optimisation

***Mutual Information Estimation and Data Discretisation***

MI-based methods use entropy estimators to infer pairwise interactions between genes. In this study, all computation for MI-based methods were carried out using the MINET package , developed as an external library in the R programming language . MINET provides a choice among four entropy estimators to calculate MI: an empirical entropy estimator, the Miller-Madow corrected estimator, the Shrink entropy estimator, and the Schurmann-Grassberger estimator. Each of these estimators can be applied only to discrete variables. To apply them to expression microarray data presented as continuous values, a discretisation step is needed. Three discretisation methods (equal width, equal frequency, global equal width) are implemented in MINET. Discretisation must result in each bin containing a significant number of samples. The default number of bins in MINET is set to the square root of the number of samples; here we explored a wide range of bin number (2 to 95) to provide an optimum setting.

***Estimation of correlation***

As with MI-based estimators, the correlation-based methods PCIT and WGCNA use estimators to infer correlation between two genes. We used three correlation estimators in this study: Pearson, Spearman and Kendall-Tau. In addition to the correlation estimator, the WGCNA method employs a soft threshold, called softpower, to emphasise large correlations at the expense of low correlations. Most previous applications of WGCNA have used softpower setting in the range from 10 to 15; here we explored softpower thresholds from 7 and 17.

**Parameter value** **optimisation–workflow**

For parameter value optimisation we used a DREAM 4 Multifactorial dataset with 100 genes and 100 samples. For RN, MRNET, CLR and ARACNE we examined three parameters: MI estimators, discretisation methods and bin size. As MI cannot be calculated directly, we optimized four different MI estimators: mi.empirical, mi.mm, mi.shrink and mi.sg (see above). Each of these methods applies to discrete values, so data discretisation is required; therefore for each MI estimator we examined three discretisation methods: equal frequency, equal width, global equal width and for each discretisation method we varied bin number from 2 to 95 (for 2-10 with increment 1, and thereafter with increment 5). Thus in total, for each method we examined 312 parameters values (4 MI estimators × 3 data discretisation methods × 26 bin sizes). For PCIT, WGCNA and CORRELATIONS we examined three correlation estimators: Pearson, Spearman and Kendall-Tau. In addition, for WGCNA we examined softpower thresholds between 7 and 17. For each of PCIT, CORRELATIONS and WGCNA we examined 3, 3, and 33 parameters values respectively. All calculations were carried out using R and Python scripts run on The University of Queensland’s high-performance computing facility.

For RN, MRNET, CLR, ARACNE and CORRELATIONS we used the MINET package implemented in R, whereas PCIT, WGCNA and GENIE were available as independent R packages. The first step in the automated process involved network inference, followed by post-processing of inferred networks to remove target-target interactions (*i.e.* those for which the source gene is not a transcription factor); this yields exclusively transcription-factor target interactions. The final step involved calculation of AUC using the Python script. Prediction accuracies for each method and parameter-value combination were written to a file in descending order, making it possible to extract the parameter combination with maximum AUC for each method. Results of parameter optimisation are presented below.

**Results**

For RN, the best MI estimator was mi.mm whereas mi.empirical was the best for MRNET, CLR and ARACNE. The best data discretisation method for all these methods was equal width with four bins, except equal frequency with five bins for ARACNE. Although differences in median accuracy among these methods are small, RN with mi.mm, equal width and four bins is the best MI-based method. We found that bin numbers between three and six give optimum performance irrespective of combination of any GRNI, MI estimator and discretisation method (Figure S1). For PCIT, WGCNA and CORRELATIONS, Pearson was the best correlation estimator, while varying the softpower value in WGCNA did not significantly affect inference accuracy.

**Supplemental Figures**

Supplemental Figure S1 Prediction accuracies of MI based methods on the multifactorial DREAM4 data for all the parameter values investigated. Irrespective of the MI estimators and discretisation methods, the best accuracies were obtained for bin-size range of 3 to 6. For the remaining methods, differences in the accuracies due to parameter variation were negligible.

Supplemental Figure S2 Prediction accuracies of methods on 12 different datasets generated from three different source networks *E. coli* large, *S. cerevisiae* and *E. coli* small. Properties of each source network are given in Table 1. Each sub-graph shows the comparison of methods using accuracies obtained with optimal parameter values on individual datasets. Overall accuracies are better on the sub-networks obtained from the *E. coli* small source network, followed by those from *S. cerevisiae*.

**Supplemental Tables**

Supplemental Table S1 Summary of GRNI methods and the parameters used in the study. Parameter values used for each parameter are given in the text above.

| **Method Abbreviations** | **Methods** | **Approach** | **Parameters used** |
| --- | --- | --- | --- |
| RN | Relevance Networks | MI | MI estimator, data discretisation method, number of bins |
| MRNET | Minimum Redundancy Networks | MI | MI estimator, data discretisation method, number of bins |
| CLR | Context-likelihood Relatedness | MI | MI estimator, data discretisation method, number of bins |
| ARACNE | The Algorithm for the Reconstruction of Accurate Cellular Networks | MI | MI estimator, data discretisation method, number of bins |
| PCIT | Partial Correlation and Information Theory | Partial correlation and MI | Correlation estimator |
| WGCNA | Weighted Gene Co-expression Network Analysis | Correlation | Correlation estimator and softpower |
| CORRELATIONS | Simple correlations | Correlation | Pearson, Spearman and Kendall-Tau |
| GENIE3 (referred as GENIE) | GEne Network Inference with Ensemble of trees | Regression | None |
| SIRENE | Supervised Inference of Regulatory Networks | Support vector machine | None |

***Supplimental Table S2*** *Parameter settings used for simulated dataset generation using SynTReN.*

| **Parameter** | **Values** |
| --- | --- |
| Burn-in_period | 1000 |
| nr_experiments | 100 |
| nr_samples_per_experiment | 1 |
| nr_nodes | 100 |
| nr_background_nodes | 0 |
| probability_for_complex_2-regulator_interactions | 0.3 |
| biological_noise | 0.1 |
| experimental_noise | 0.1 |
| noise_on_correlated_inputs | 0.0 |
| nr_external_nodes | -1 |
| nr_correlated_external_nodes | -1 |
| subnetwork_selection_method | cluster |
| source_network | *E.* *coli* small, *E. coli* large and *S.* *cerevisiae* |
| random_seed | 13 |

Supplemental Table S3 Details of datasets used in the study.

| **Dataset** | **Number of genes/nodes** | **Number of samples** |
| --- | --- | --- |
| DREAM3  (Knockdown) | 100 | 100 |
| DREAM4  (Multifactorial) | 100 | 100 |
| SynTReN *E. coli* small | 10, 50, 100, 200 | 50, 100, 200 |
| SynTReN *E. coli* large | 10, 50, 100, 200 | 50, 100, 200 |
| SynTReN *S. cerevisiae* | 10, 50, 100, 200 | 50, 100, 200 |
| Ovarian cancer  microarray | 2452 and 282 | 12 |

Supplemental Table S4 Topological properties of source networks used to generate synthetic expression data using SynTReN.

| Network properties | ***E. coli* large** | ***E. coli* small** | ***S. cerevisiae*** |
| --- | --- | --- | --- |
| Number of nodes | 1330 | 423 | 690 |
| Number of interactions | 2774 | 578 | 1094 |
| Average directed path | 1.85 | 1.36 | 1.44 |
| Average clustering coefficient | 0.20 | 0.085 | 0.047 |

Supplemental Table S5 Parameter settings used for normal and ovarian cancer network generation using SIRENE.

| **Parameter** | **Value** |
| --- | --- |
| kernelType | rbf |
| method | libsvm |
| C | 1000 |
| param | 1.2247 |
| nsplitPredict | 3 |
| nSplitCv | 10 |
| defaultPositiveScore | 100 |
| rocCurveLength | 1000 |

Supplemental Table S6 Optimal parameter setting and corresponding accuracy obtained for each method on five different datasets with 100 genes and 100 samples. mm= mi.mm, emp=mi.empirical, sh=mi.shrink, sg=mi.sg, ef=equal frequency, ew=equalwidth, gew=globalequalwidth.

‘+’ indicates parameter values= mutual information estimator+data discretization method+number of bins.

|  | **DREAM4**  **Multifactorial** | | **DREAM3**  **Knockdown** | | ***E. coli* small** | | ***E. coli* large** | | ***S. cerevisiae*** | |
| --- | --- | --- | --- | --- | --- | --- | --- | --- | --- | --- |
|  | **Parameter**  **Values** | **AUC** | **Parameter**  **Values** | **AUC** | **Parameter**  **Values** | **AUC** | **Parameter**  **Values** | **AUC** | **Parameter**  **Values** | **AUC** |
| RN | mm+ew+4 | 0.75 | mm+gew+9 | 0.58 | sh+ef+5 | 0.83 | sh+ew+2 | 0.63 | sg+ew +5 | 0.85 |
| MRNET | emp+ew+4 | 0.73 | mm+gew+9 | 0.60 | sg+ew+10 | 0.87 | sh+ef+8 | 0.67 | sg+ew+10 | 0.89 |
| CLR | emp+ew+4 | 0.74 | mm+ew+3 | 0.58 | sg+ew+10 | 0.86 | mm+ef+8 | 0.66 | sg+ew+8 | 0.88 |
| ARACNE | emp+ef+5 | 0.64 | sg+gew+6 | 0.54 | emp+gew+7 | 0.63 | emp+ef+10 | 0.55 | sg+ew+10 | 0.70 |
| PCIT | Spearman | 0.52 | Spearman | 0.52 | Kendall | 0.78 | Spearman | 0.60 | Kendall | 0.82 |
| WGCNA | Pearson | 0.75 | Pearson | 0.55 | Kendall | 0.83 | Kendall | 0.58 | Kendall | 0.83 |
| CORRELATION | Pearson | 0.75 | Pearson | 0.55 | Kendall | 0.81 | Kendall | 0.57 | Kendall | 0.83 |

Supplemental Table S7 Mann-Whitney U test p-values calculated for each method using the accuracies obtained at optimum parameter setting for 36 datasets from three source networks.

| **Method** | **E. coli** small &  *E. coli* large | *S. cerevisiae* &  *E. coli* large | E. coli ***small* &**  S. cerevisiae |
| --- | --- | --- | --- |
| RN | 1.82e-05 | 1.75e-05 | 2.00e-02 |
| MRNET | 1.82e-05 | 1.82e-05 | 3.00e-02 |
| CLR | 1.82e-05 | 1.82e-05 | 3.00e-03 |
| ARACNE | 1.23e-03 | 1.47e-02 | 1.30e-01 |
| PCIT | 2.34e-05 | 1.82e-05 | 3.30e-01 |
| WGCNA | 1.82e-05 | 1.82e-05 | 2.00e-03 |
| GENIE | 1.82e-05 | 1.81e-05 | 4.00e-03 |
| CORRELATION | 1.82e-05 | 1.82e-05 | 1.30e-01 |

**Supplemental Table S8** Top 10 interactions from normal and cancer network predicted by SIRENE. Please see the text for proposed roles and mechanisms of action for interactions between E2F1 with DKK1, and between E2F1 and HSD17B2.

| **Transcription**  **Factor** | **Target**  **Gene** | **Weight in**  **Normal** | **Weight in**  **Cancer** | **Target**  **Gene Function** | **Matrix**  **Similarity Score** |
| --- | --- | --- | --- | --- | --- |
| SP3 | NPY1R | 1.08 | -0.23 | GPCR | No TFBS |
| SP3 | AGA | 0.75 | -0.4 | Enzyme | 0.96 |
| NFKB1 | BCHE | 0.75 | -0.38 | Enzyme | No TFBS |
| E2F1 | HBB | 0.69 | 0.27 | Transporter | No TFBS |
| E2F1 | CDK7 | 0.52 | -0.19 | Enzyme | 0.76 |
| SP3 | NTNG1 | 0.52 | -0.37 | Receptor ligand | 0.88 |
| E2F1 | DKK1 | 0.49 | -1.03 | Receptor Ligand | 0.89 |
| E2F1 | CCR7 | -0.19 | 0.81 | GPCR | 0.71 |
| NFKB1 | TPI1 | -0.37 | 0.57 | Enzyme | 0.86 |
| E2F1 | HSD17B2 | -0.43 | 0.59 | Enzyme | 0.81 |

Supplemental Table S9 Angiogenesis-specific target genes, with evidence for their involvement in angiogenesis. These target genes, predicted to be regulated by SP3 or NFκB1 in normal cells, are inferred to be regulated by E2F1 in ovarian cancer instead.

| **Gene** | **References in angiogenesis (Pubmed ID)** |
| --- | --- |
| CAV1 | 21830216 |
| ADAMTS3 | 15073121 |
| DACH1 | 20937839 |
| TIMP3 | 16644110 |
| NPY | 20508839 |
| NPY1R | 17979777 |
| CALB2 | 17148947 |
| LAMB1 | 21896617 |
| DPYD | 11099324 |
| KDR | 17396134 |
| FGF13 | 21436589 |
| FGL2 | 18932275 |
| AOX1 | 17632767 |
| PRRX1 | 16373852 |
| PAPSS2 | 15326482 |
