# Supplementary figures and images for: Gene regulatory network inference: evaluation and application to ovarian cancer allows the prioritization of drug targets
Source: Genome Med. 2012 May 1;4(5):41. doi: 10.1186/gm340 (PMC3506907; doi:10.1186/gm340)

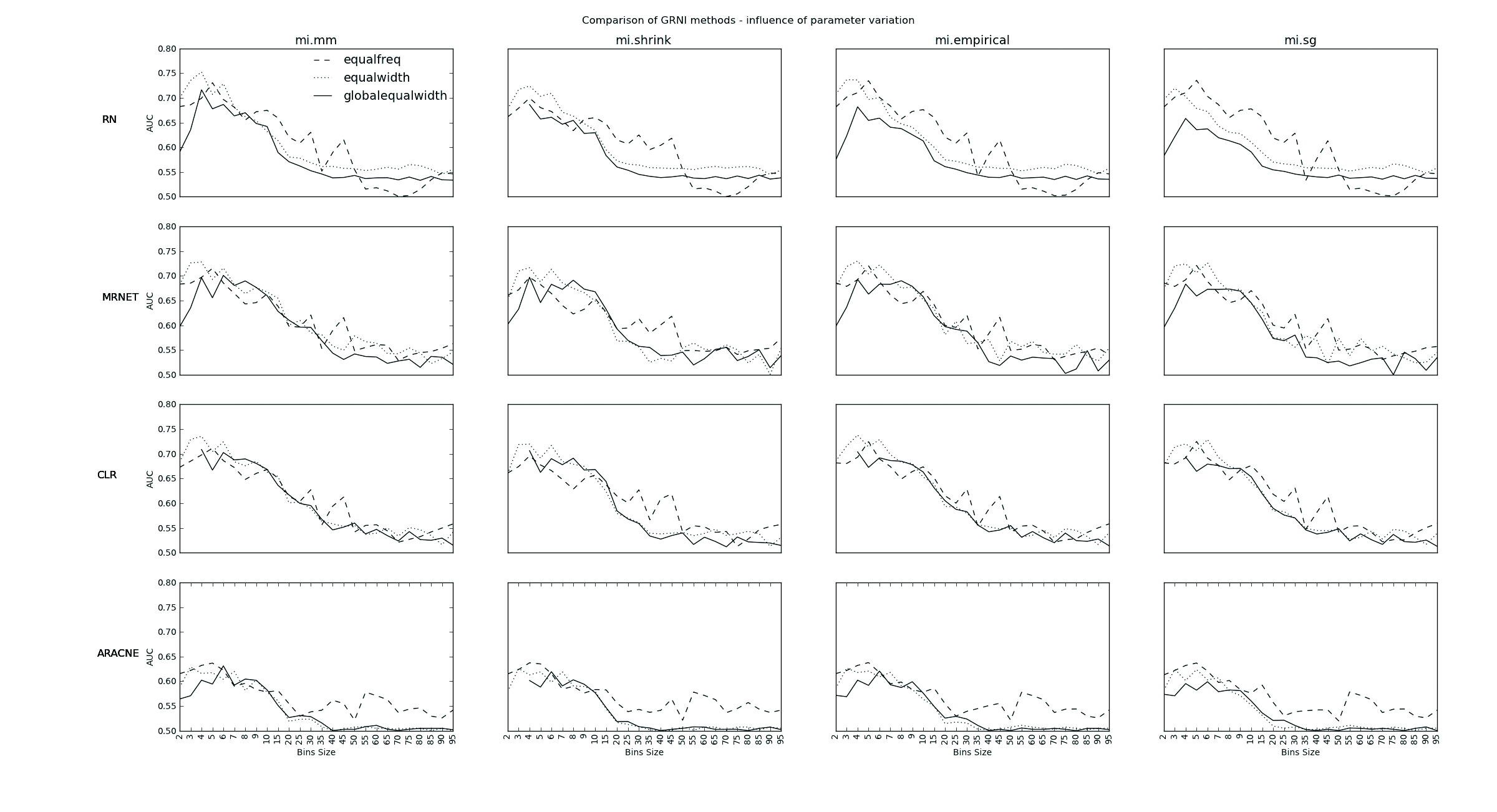

Supplement: Additional file 3 — Figure S1 - prediction accuracies of MI-based methods on the multifactorial DREAM4 data. Prediction accuracies of MI based methods on the multifactorial DREAM4 data for all the parameter values investigated. [file gm340-S3.TIFF]

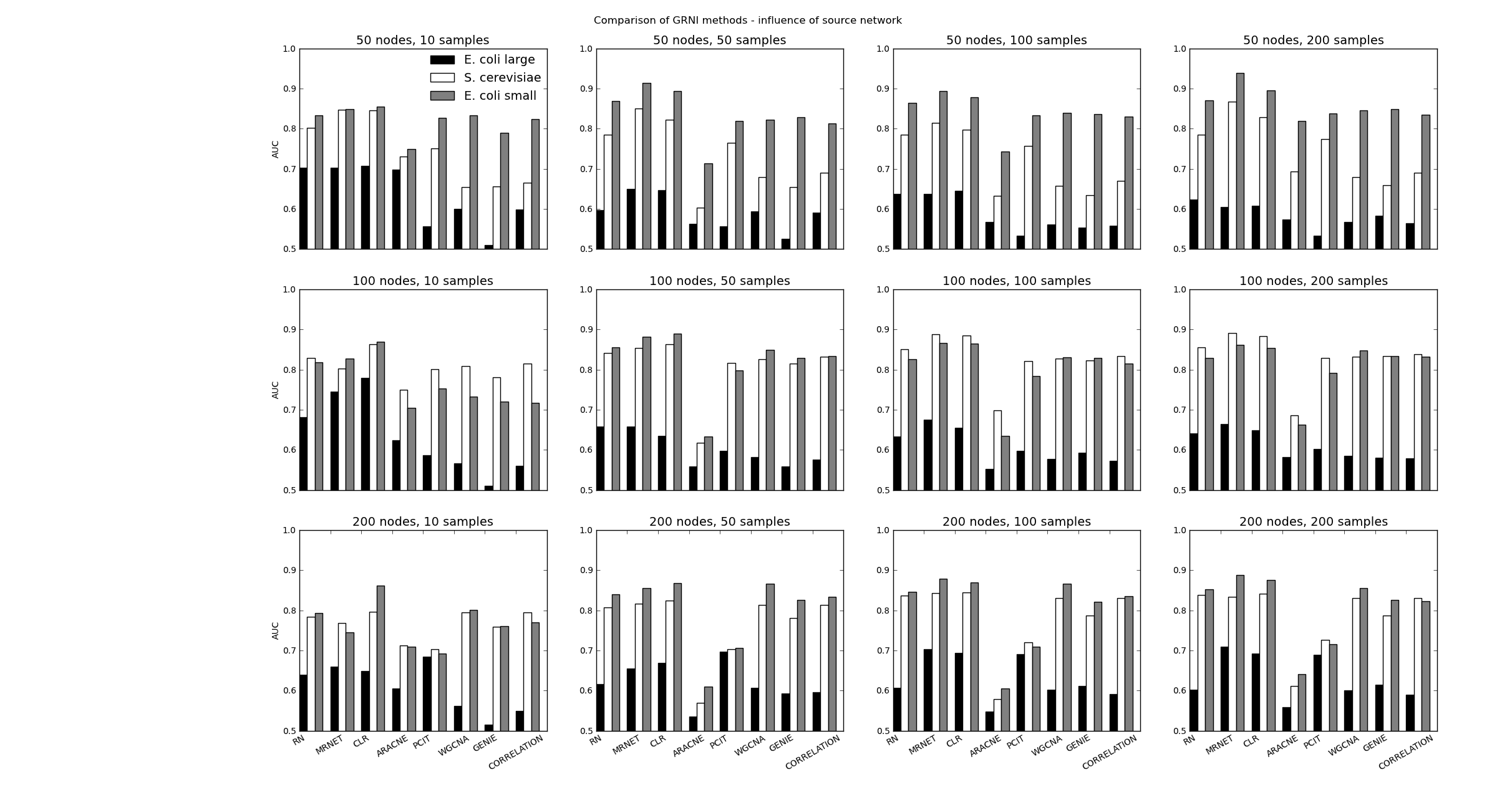

Supplement: Additional file 4 — Figure S2 - prediction accuracies of methods on datasets generated from three different source networks. Prediction accuracies of methods on 12 different datasets generated from three different source networks: E. coli large, S. cerevisiae and E. coli small. [file gm340-S4.TIFF]
